# Melanin-Inspired Biomimetic Strategy for Preserving Adhesion of Lubricants via Thiol-Quinone Addition

**DOI:** 10.3390/biomimetics11040269

**Published:** 2026-04-14

**Authors:** Xiao Song, Chao Mei, Yinna Wu, Dan He, Junwei Zhu, Qi Chen, Jiaxin Guo, Zhengwei Zhao, Tonghui Xie, Wenbin Liu

**Affiliations:** 1 School of Chemical Engineering, Sichuan University, Chengdu 610065, China; 2 Exploration Division, Southwest Oil & Gas Field Company, PetroChina, Chengdu 610041, China; 3 Oil & Gas Technology Research Institute, Southwest Oil & Gas Field Company, PetroChina, Chengdu 610017, China

**Keywords:** biomimetic synthesis, melanin-inspired, thiol-quinone addition, protection of catechol, high-adhesion lubricants, drilling fluid

## Abstract

Lubricants are essential for water-based drilling fluids. Catechol-based lubricants provide improved lubrication performance owing to their strong adhesion ability through the formation of coordination bonds inspired by mussel adhesion. However, the conventional synthetic ester and amide lubricants suffer from loss of adhesive capability due to hydrolysis and autoxidation. Inspired by mussels and melanin biosynthesis, a biomimetic strategy was developed to synthesize a high-adhesion lubricant with good stability via thiol-quinone Michael addition to restore and stabilize the catechol moiety. Bisphenol A was oxidized to the corresponding quinone using 2-iodoxybenzoic acid. Subsequent Michael addition reaction with 1-octadecanethiol produced a thiol-functionalized lubricant containing catechol moieties and long alkyl chains through an *S*-catecholyl linkage. Biomimetic principles were incorporated into both the molecular structure and the synthetic route, emulating the structural and functional features of mussel adhesion and melanin biosynthesis. Octadecanethiol provided sulfur-containing extreme-pressure functionality and contributed to strong adsorption on metal surfaces. The molecular structure was confirmed by FTIR, ^1^H NMR, and ^13^C NMR. The thiol-functionalized lubricant formed strong coordination with Fe^3+^ and Fe^2+^ ions across a wide pH range, with an apparent complexation stoichiometry of 1:1 and conditional stability constants of 4.09 and 5.02, respectively. Bis-coordination formed a cross-linking network. It exhibited good resistance toward autoxidation and thermal stability up to 350 °C. In bentonite-based drilling fluids, the extreme pressure lubrication coefficient and adhesion coefficient at a 1% addition were 0.06 and 0.07, respectively. The coefficient of friction and wear scar diameter were 0.09 and 0.63 mm, respectively. The increased contact angle confirmed strong adsorption of the lubricant on metal surfaces. The lubricant combined strong adhesion, high stability, and excellent compatibility with drilling fluids, highlighting its potential as an advanced biomimetic lubricant. This biomimetic thiol-quinone addition strategy provides an effective approach to overcome the instability of conventional catechol-based lubricants.

## 1. Introduction

As the “lifeblood of petroleum engineering”, drilling fluids play a crucial role in reducing friction and torque within the wellbore during drilling operations. Water-based drilling fluids are more environmentally friendly and cost-effective; however, their inadequate lubricity significantly limits their wider application. To address this issue, lubricants are commonly added to improve lubrication performance. The long-chain hydrocarbon segments in these lubricants can form a compact hydrophobic lubricating film on the frictional contact surface, thereby reducing shear stress [1,2]. Conventional lubricants exhibit weak adsorption at the interface. In practical downhole environments, the complex aqueous conditions and the shearing action of other additives in water-based drilling fluids may lead to lubricant detachment from the surface when its adhesion is insufficient. This impedes the formation of an effective lubricating film between the drill string and the wellbore, substantially compromising lubrication performance [3].

In nature, the blue mussel (*Mytilus edulis*) can firmly adhere to various wet and dynamic solid surfaces in the turbulent intertidal zone by secreting adhesive mussel foot proteins (Mfps). The molecular basis of this remarkable adhesion is attributed to the ortho-dihydroxyphenyl (catechol) moiety of the noncanonical amino acid L-3,4-dihydroxyphenylalanine (DOPA) residues abundant in these priming proteins, which can bind to a variety of substrate surfaces through complex intermolecular interactions, including hydrogen bonding, bidentate metal coordination, and π-π interactions. The remarkable adhesive properties of mussel adhesive plaque have inspired the development of diverse biomimetic materials containing DOPA-like structures over the past decades [4,5,6]. Previous studies reported that a DOPA-inspired biomimetic lubricant was synthesized through the esterification of dihydroxybenzoic acid and oleyl alcohol, where catechol groups were introduced into the molecular structure, thus endowing the lubricant with enhanced adhesive performance [3].

However, conventional ester- and amide-based lubricants exhibit poor resistance to hydrolysis and high temperatures [7]. Hydrolysis can lead to foaming and corrosion, posing significant risks to drilling safety. More importantly, the autoxidation susceptibility of the catechol moiety inevitably leads to a loss of adhesive capability [8,9]. The autoxidation propensity limits the interfacial adhesion of these materials in aqueous environments. The catechol must resist autoxidation to maximize the bidentate coordination bonding. Therefore, improving the stability of catechol groups represents a critical breakthrough for the practical application of catechol-containing biomimetic lubricants [10]. However, limited research has focused on protecting susceptible catechol groups from autoxidation in biomimetic lubricants in order to preserve their adhesive performance. Therefore, rational molecular design is required to preserve the structural integrity of the catechol moiety.

Nature also provides inspiration to address the above-mentioned challenges. Mussels circumvent catechol autoxidation by utilizing the cysteine-rich protein Mfp-6 as a sacrificial reductant that restores DOPA through thiol-mediated reduction of dopaquinone [8,9,11,12]. The versatile chemistry of the catechol enables diverse covalent conjugation reactions. The catechol is readily oxidized to yield highly reactive o-quinone. o-Quinones readily react with thiol nucleophiles to generate thiol-catechol adducts through Michael-type addition reactions [13]. The catechol oxidation and thiol-quinone addition reaction are central to a variety of biological processes, including pigmentation, neurotransmission, wound healing, insect cuticle sclerotization, byssal thread adhesion, squid ink formation and beak hardening, and sandcastle worm tube-building [14]. Recently, thiol-quinone addition has been increasingly exploited as an efficient strategy to introduce adhesive functionalities with thiol-catechol connectivity [13,15,16]. It has been reported that conjugation of nucleophilic groups to catechol can enhance adhesion performance [8,12]. In this work, we propose the first attempt to address the challenge by simultaneously utilizing both catechol- and thiol- chemistry found in mussel adhesive proteins. The resulting adduct effectively preserves the ortho-dihydroxyphenyl functional groups, thereby maintaining strong adhesive capability.

**Scheme 1:**
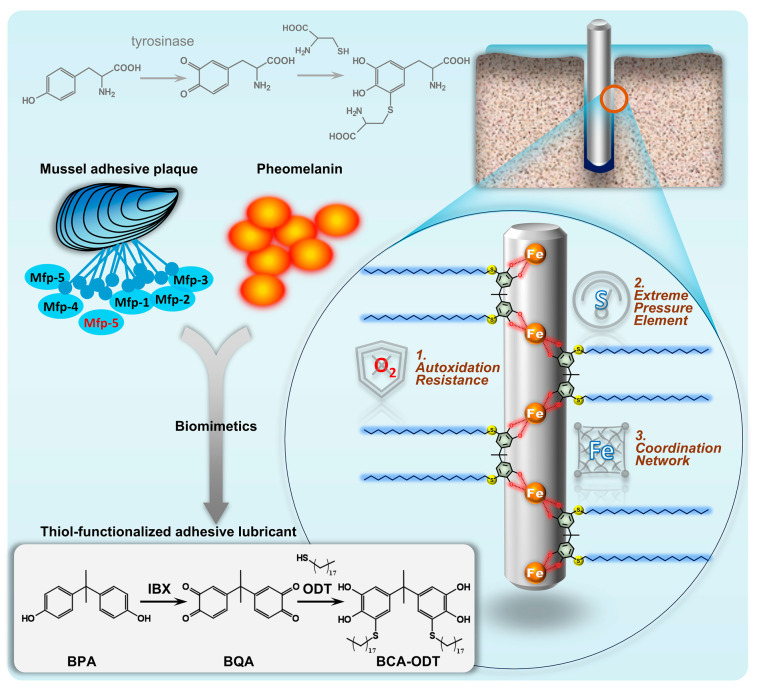
Bioinspiration from melanin for thiol-functionalized biomimetic lubricant.

Michael 1,6-addition of thiols to o-quinones, best exemplified by the biologically relevant conjugation of cysteine to dopaquinone in melanin biosynthesis, exhibits extremely rapid kinetics and distinct regioselectivity for the C5 position [14]. Melanin, a naturally occurring biomacromolecule, is synthesized when tyrosinase first hydroxylates tyrosine to form DOPA and subsequently oxidizes DOPA to the electrophilic intermediate dopaquinone [17]. In the eumelanin pathway, dopaquinone undergoes intramolecular cyclization with its pendant amino group to form dopachrome. In the pheomelanin pathway (Scheme 1), in the presence of cysteine, dopaquinone preferentially reacts with the thiol group of cysteine through a Michael 1,6-addition to form 5-*S*-cysteinydopa [18,19,20]. Thus, the cysteinyldopa structure contains both a catechol group and an aryl thioether linkage.

Accordingly, inspired by mussel adhesion and melanin biosynthesis, a novel synthetic route to a biomimetic lubricant was designed based on thiol-quinone coupling chemistry (Scheme 1). Bisphenol A (BPA) was first oxidized to bisquinone A (BQA) using 2-iodoxybenzoic acid (IBX), a commonly employed oxidant in organic chemistry capable of converting monophenols into corresponding o-quinones under mild conditions, as reported by Börner’ s pioneering works [15,16]. Subsequently, taking advantage of the high reactivity of the quinone moiety, BQA readily reacted with the thiol nucleophile 1-octadecanethiol (ODT) through a Michael addition reaction to covalently conjugate the long alkyl chain onto the aromatic framework via an aryl thioether linkage, accompanied by reduction of BQA back to its original biscatechol A (BCA) structure. Therefore, the catechol structure was efficiently restored and protected from oxidation. As a result, a high-adhesion lubricant containing *S*-catecholyl functionality was obtained. This strategy effectively preserves the catechol structure and minimizes the risk of autoxidation, thereby enhancing durable adhesion. The chemical structure of the synthesized product was confirmed by FTIR, ^1^H NMR, and ^13^C NMR spectroscopy. In addition, the performance of the lubricant was evaluated by investigating its tribological properties, thermal stability, wettability, and rheological behavior.

Biomimetic principles were incorporated into both the molecular structure and the synthetic route, emulating the characteristics, functions, and mechanisms of mussel adhesion and melanin biosynthesis. The proposed thiol-functionalized biomimetic lubricant embodies its bionic concepts in four aspects: namely (i) protection of catechol groups from autoxidation through the formation of *S*-catecholyl functionalities inspired by mussel Mfp-6 protein; (ii) strong interfacial adhesion derived from catechol groups inspired by mussel adhesive chemistry; (iii) a green synthetic route based on spontaneous thiol-quinone addition reaction inspired by melanin biosynthesis; and (iv) one-pot oxidation of BPA to o-quinone using IBX, mimicking the tyrosinase-catalyzed oxidation involved in melanin biosynthesis. Consequently, the resulting lubricant more effectively mimics the adhesion mechanism of mussels. Octadecanethiol acts as an efficient nucleophile that readily reacts with o-quinone to form the corresponding adduct. Octadecanethiol provides both a long hydrophobic chain and sulfur-containing extreme-pressure functionality [21,22]. Octadecanethiol also contributes to strong adsorption and defoaming on the metal surface [7]. Due to the facile fabrication process and inexpensive raw materials, the synthesis of the thiol-functionalized biomimetic lubricant is expected to be cost-effective and scalable. This study provides new insight into the molecular design strategy for durable catechol-based lubricants with enhanced resistance to autoxidation.

## 2. Materials and Methods

### 2.1. Materials

All reagents used in this study were purchased from Aladdin Industrial Co., Ltd. (Shanghai, China), including bisphenol A (BPA), 2-iodoxybenzoic acid (IBX, 80%, stabilized with benzoic acid and isophthalic acid), and 1-octadecanethiol (ODT).

### 2.2. Preparation of Lubricant

BPA (228.29 mg, 1 mmol) was dissolved in methanol (10 mL), followed by the addition of IBX (840.06 mg, 3 mmol). The mixture was magnetically stirred in a water bath at 35 °C for 10 min. After the reaction, the mixture was cooled in an ice bath for 10 min and subjected to vacuum filtration, and the solid was air-dried at room temperature. The crude product was redissolved in dichloromethane and centrifuged to remove insoluble impurities. The supernatant was concentrated under reduced pressure to afford BQA as a solid.

BQA (51.25 mg, 0.2 mmol) was dissolved in dichloromethane (15 mL), while ODT (114.62 mg, 0.4 mmol) was dissolved in dichloromethane (5 mL). The two solutions were mixed and stirred in a water bath at 25 °C for 2 h. The reaction was conducted under a nitrogen atmosphere to prevent the formation of disulfide bonds between thiol groups. After the reaction, the mixture was concentrated under reduced pressure to afford a crude product, which was purified by column chromatography.

The crude product was purified by silica gel column chromatography (100–200 mesh) using petroleum ether/ethyl acetate as the eluent with a gradient from 100:1 to 5:1. When the ratio of petroleum ether/ethyl acetate reached 10:1, the target product fraction appeared, and the elution was continued with the same solvent ratio of 10:1. The fractions containing the target product were collected and concentrated under reduced pressure to afford the purified product. The thiol-functionalized biomimetic lubricant obtained in this study is denoted as BCA-ODT.

Fourier transform infrared (FTIR) spectra were recorded on a PerkinElmer Spectrum Two spectrometer (Llantrisant, UK) using the KBr pellet method. Each sample was scanned three times over the wavenumber range of 4000–400 cm^−1^. Nuclear magnetic resonance (NMR) spectra were recorded on a JEOL JNM-ECZ400S/L1 nuclear magnetic resonance spectrometer (Tokyo Japan), operating at 400 MHz for ^1^H NMR and 101 MHz for ^13^C NMR. BPA and BQA were dissolved in dimethyl sulfoxide-d6 (DMSO-d6), while ODT and BCA-ODT were dissolved in chloroform-d (CDCl_3_).

### 2.3. Contact Angle Measurements

Ethanolic solutions (5 mM) of BCA-ODT were prepared. Iron, copper, aluminum alloy, steel and glass (SiO_2_) plates (1 × 1 cm, 0.3 mm thickness) with relatively smooth and flat surfaces were used as substrates. Before treatment, all plates were cleaned with ethanol and then oven-dried. The substrates were separately immersed in the BCA-ODT solution, and the contact angles of deionized water on the treated metal surfaces were subsequently measured using an Shengding SDC-100 contact angle goniometer (Dongguan, China).

### 2.4. Quantitative Analysis for Interaction with Metal Ions

#### 2.4.1. Job’s Method

A 10 mM ferric chloride (FeCl_3_) ethanol solution, 10 mM ferrous chloride (FeCl_2_) ethanol solution, and 1 mM BCA-ODT ethanol solution were prepared. The solutions were mixed to obtain a final concentration of 200 μM. The molar ratio of metal ions to BCA-ODT ($q=\frac{\left[{Fe}^{3+}/{Fe}^{2+}\right]}{\left[{Fe}^{3+}/{Fe}^{2+}\right]+\left[BCA-ODT\right]}$) was varied starting from 0 to 1 in increments of 0.1. After incubation for 1 h to allow the reaction to reach equilibrium, the UV-Vis absorption spectra of the solutions were recorded using a Mapada UV-3100PC spectrophotometer (Shanghai, China) equipped with a quartz cuvette of 10 mm path length.

#### 2.4.2. Determination of K_cond_

The molar ratio of metal ions to BCA-ODT ($q=\frac{\left[{Fe}^{3+}/{Fe}^{2+}\right]}{\left[BCA-ODT\right]}$) was fixed at 1:1, and mixed solutions with final concentrations of 40, 80, 120, 160, and 200 μM were prepared accordingly. After incubation for 1 h to allow the system to reach equilibrium, UV-Vis absorption spectra were recorded. The experimental data were analyzed using a modified Benesi–Hildebrand (B-H) equation, and fitted according to Equation (1) to obtain the conditional stability constants (Kcond) of the complexes [23].

(1) $${\left[M\right]}_{0}={\left(\frac{\Delta A}{\Delta \varepsilon \cdot K}\right)}^{1/2}+\frac{\Delta A}{\Delta \varepsilon }$$

where [*M*]_0_ is the initial concentration of metal ions, expressed in mol/L. *ΔA* is the difference between the absorbance of the complexed mixture (*A_mix_*) and the absorbance of the individual components prior to complexation (i.e., the metal ion *A_M_* and ligand *A_L_*), as defined by Equation (2). *ε_MLn_*, *ε_M_*, and *ε_L_* correspond to the molar absorptivity of ML_n_, M, and L, respectively. *Δε* represents the difference in molar absorptivity and can be calculated according to Equation (3). K denotes the conditional stability constant of the complex.

(2) $$\Delta A=A_{mix}-A_{M}-A_{L}$$

(3) $$\Delta \varepsilon =\varepsilon_{{ML}_{n}}-\varepsilon_{M}-\frac{1}{n}\varepsilon_{L}$$

### 2.5. Autoxidation Resistance

A 1 mM ethanolic solution of BCA-ODT was prepared, and air was continuously bubbled through the solution using an air pump. Aliquots were withdrawn at predetermined time intervals over 24 h to monitor the spectral changes by UV-vis spectroscopy. The collected samples were then reacted with Fe^3+^ ions to evaluate the coordination behavior after oxidation, and the resulting spectral changes were also analyzed by UV-vis spectroscopy. The water contact angles were subsequently measured according to the method described in Section 2.3.

### 2.6. TGA-DSC

Simultaneous thermogravimetric analysis and differential scanning calorimetry (TGA-DSC) was performed using a Mettler Toledo TGA/DSC thermal analyzer (Zürich, Switzerland). An appropriate amount of each sample was placed in an alumina crucible and analyzed under a nitrogen atmosphere. Measurements were carried out over a temperature range of 30–900 °C at a heating rate of 10 °C/min.

### 2.7. Evaluation on Lubrication Performance

#### 2.7.1. Preparation of Freshwater-Based Slurry

To prepare the 4 wt% freshwater-based bentonite slurry, 500 mL of deionized water was placed in a beaker, followed by the addition of 0.8 g of anhydrous Na_2_CO_3_ and 20 g of sodium bentonite. The mixture was stirred at 10,000 rpm for 20 min, then sealed and aged at room temperature for 24 h.

#### 2.7.2. Lubricity Coefficient Test

A lubricant dosage of 1 wt% was added to the bentonite-based slurry. An Coriolis EP lubricity tester (Houston, TX, USA) was used to measure the extreme-pressure lubricity coefficient. The lubricity coefficient and reduction rate were calculated using Equations (4) and (5).

(4) $$K_{f}=\frac{K_{d}}{100}\times \frac{34}{K_{\omega }}$$

where *K_f_* is the lubricity coefficient, *Kω* is the torque reading for water, *K_d_* is the torque reading corresponding to the drilling fluid during testing, and 34 is the correction factor.

(5) $$\Delta K_{f}=\frac{K_{0}-K_{f}}{K_{0}}\times 100\%$$

where *K*_0_ is the lubricity coefficient of the base slurry, *K_f_* is the lubricity coefficient after adding lubricant to the base slurry, and *ΔK_f_* is the lubricity coefficient reduction rate.

#### 2.7.3. Adhesion Coefficient Test

The mud cake adhesion coefficient is used to evaluate the lubricity of drilling fluid mud cakes and the static friction between the mud cake, drill collar, and wellbore wall. The adhesion coefficient was measured using an OFI 150-50 adhesion coefficient tester (Houston, TX, USA). The reduction rate of the adhesion coefficient was calculated using Equation (6):

(6) $$\Delta K_{a}=\frac{K_{a,0}-K_{a}}{K_{a,0}}\times 100\%$$

where *K_a_*_,0_ is the adhesion coefficient of the base slurry, *K_a_* is the adhesion coefficient after adding lubricant to the base slurry, and *ΔK_a_* is the adhesion coefficient reduction rate.

#### 2.7.4. Four-Ball Friction Tester and Surface Morphology Analysis

To further evaluate the lubricating performance of the lubricant under simulated point-contact friction conditions, four-ball friction tests were performed using sodium bentonite freshwater base slurry containing 1 wt% BCA-ODT as the test sample. Friction tests were performed using an Hengxu SGW-10A four-ball friction tester (Jinan, China) with 12.7 mm GCr15 bearing steel balls under a load of 300 N (corresponding to a maximum Hertz contact pressure of approximately 3.15 GPa) and a rotational speed of 300 rpm for 20 min. The steel balls were cleaned by ultrasound and oil cups was washed using petroleum ether and absolute ethanol. The experiments were conducted at room temperature. The coefficient of friction was recorded in real time. After the test, the wear scars on the steel balls were observed using a Seepack KE-310B CMOS camera (Shenzhen, China) and analyzed, and the anti-wear performance of the lubricant was assessed by the wear scar diameters.

#### 2.7.5. Rheological Performance

Drilling fluid slurries were prepared by adding 0.5, 1, or 2 g of BCA-ODT to 100 mL of the aged base slurry, corresponding to concentrations of 0.5 wt%, 1 wt%, and 2 wt%. The mixture was stirred at 10,000 rpm for 10 min, resulting in drilling fluid slurries with mass concentrations of 0.5%, 1%, and 2%. Rheological properties were measured using a Thermo Fisher HAAKE MARS iQ Air rotational rheometer (Karlsruhe, Germany) at 25, 40, 60, and 80 °C with shear rates ranging from 0 to 500 s^−1^. The shear stress and viscosity as a function of shear rate were recorded. The data were fitted using the Bingham model. The constitutive equation of a Bingham plastic fluid is given by Equation (7):

(7) $$\tau =\tau_{0}+\mu_{p}\gamma$$

where *τ*_0_ represents the yield stress (Pa), *μ_p_* denotes the plastic viscosity (Pa·s), and *γ* denotes the shear rate (s^−1^).

### 2.8. Replicates and Data Reproducibility

Unless otherwise stated, all tests and measurements were carried out in triplicate to ensure data reproducibility.

## 3. Results and Discussion

### 3.1. Synthesis of BQA

**Figure 1:**
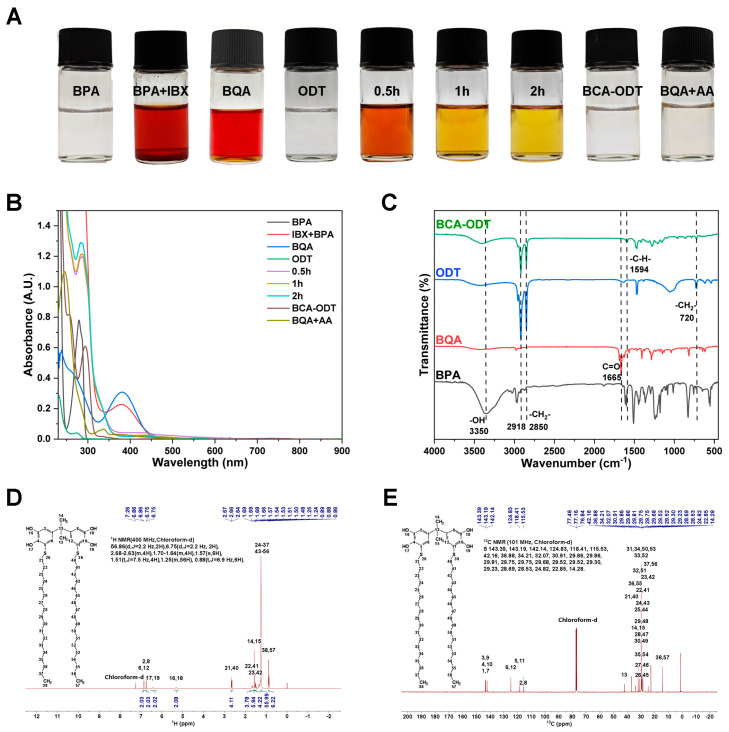
Characterization of the BCA-ODT product. (**A**) Photographs of different stages of the synthesis process. From left to right: BPA, BPA + IBX, BQA, ODT, and the product at various reaction times, 0.5 h, 1 h, and 2 h, followed by BCA-ODT and BQA + AA (addition of ascorbic acid). (**B**) UV-Vis absorption spectra. (**C**) FTIR spectra. (**D**) ^1^H NMR spectrum of BCA-ODT. (**E**) ^13^C NMR spectrum of BCA-ODT.

Upon IBX oxidation, the mixture changed from transparent to dark red, a characteristic color of catechol oxidation. A new peak emerged at 380 nm, which is the characteristic absorbance for o-quinone (Figure 1A,B). As the reaction proceeded, BQA precipitated in high yield in the form of a reddish crystal from the reaction mixture, obviating the need for further purification [15]. The crystals showed a prismatic morphology with an average length of about 140 μm (Figure S1). Disappearance of the peak at 380 nm and the hypochromic shift for the peaks at 275 nm to 245 nm upon addition of ascorbic acid can be attributed to the reduction of the o-quinone to the catechol moiety. These observations suggested that the reddish crystals were BQA formed from the oxidation of BPA.

The molecular structure of BQA was characterized by FT-IR and NMR (Figure 1C). The hydroxyl groups (–OH) stretching vibration band at 3350 cm^−1^ almost disappeared, accompanied by the emergence of a new carbonyl (C=O) stretching band at 1665 cm^−1^. BPA exhibits high molecular symmetry, with two phenolic hydroxyl protons in the downfield region at δ9.12 (Figure S2A). The aromatic protons resonated at δ6.98 and δ6.63, while the methyl protons appeared in the upfield region. ^1^H-NMR results unambiguously verified that the structure of the oxidized product was completely consistent with the predicted structure. Upon oxidation to BQA, the hydroxyl proton signals disappeared (Figure S2B). Due to partial disruption of molecular symmetry, the aromatic proton signals split into multiplets, whereas the methyl proton signals remained essentially unchanged at δ1.43. These spectral changes further confirmed the successful oxidation of BPA to BQA.

Quantitative determination of BQA was performed using the standard curve at an absorbance of 380 nm (Figure S3A). The oxidation conditions were optimized through single-factor experiments (Figure S4). A yield of 73.29% was obtained using the following optimized reaction conditions: BPA concentration of 100 mM, molar ratio of IBX to BPA of 1:3, reaction temperature of 35 °C, and reaction time for 10 min.

### 3.2. Synthesis of BCA-ODT Adduct as Thiol-Functionalized Lubricant

After the addition of ODT, the color of the mixture solution was changed immediately from dark red to pale yellow, indicating a rapid consumption of BQA [15]. The quinone peak at 380 nm vanished rapidly. A single peak was observed at 291 nm, indicating that most of the catechol moiety was retained (Figure 1A,B). These results illustrated that BQA actually reacted with ODT to synthesize BCA-ODT.

In the FT-IR spectrum of BCA-ODT (Figure 1C), the carbonyl absorption band at 1665 cm^−1^ disappeared, while the hydroxyl stretching band at 3350 cm^−1^ reappeared, indicating that the reduction of the quinone group led to the restoration of the catechol group. Owing to intramolecular hydrogen bonding within the catechol moiety, the –OH band seemed weakened. Furthermore, the characteristic symmetric and asymmetric stretching vibrations of methyl (–CH_3_) and methylene (–CH_2_–) groups at 2918 and 2850 cm^−1^, together with the in-plane rocking vibration of long methylene chains (–(CH_2_)_n_–) at 720 cm^−1^, were also observed. This indicated that the conjugation of 1-octadecanethiol onto the aromatic framework proceeded as expected.

In the ^1^H NMR spectrum of BCA-ODT (Figure 1D), a triplet at δ0.88 corresponding to the terminal methyl protons of the long alkyl chain, along with the multiplet at δ1.25 assigned to the methylene protons of the alkyl chain, were clearly observed, confirming the presence of the long hydrocarbon segment from ODT (Figure S2C). The electron-withdrawing inductive effect of the sulfur atom on the adjacent alkyl groups led to deshielding and a downfield shift. As a result, signals at δ2.68–2.63 and δ1.70–1.64 were attributed to the methylene protons in the regions adjacent to the sulfur atom. The singlet at δ1.57 was assigned to the methyl protons from BPA. In the aromatic region, the resonance at δ6.86 corresponded to protons on the benzene rings, while the peaks at δ6.75 were attributed to protons at another aromatic position. The observed upfield shift in the spectrum was attributed to shielding at the ortho-position, caused by the electron-donating conjugative effect of the sulfur atom on the adjacent benzene ring. The four phenolic hydroxyl protons appeared as weak broad signals at about δ6.5 and δ5.5, which can be attributed to proton exchange with the deuterated solvent or intramolecular hydrogen bonding. These spectral features confirmed the successful Michael addition of ODT to BQA, resulting in the restoration of the catechol structure.

BQA: ^1^H NMR (400 MHz, DMSO-d6) δ7.08 (dd, J = 10.3, 2.6 Hz, 2H), 6.40–6.33 (m, 4H), 1.43 (s, 6H).
ODT: ^1^H NMR (400 MHz, Chloroform-d) δ2.51 (q, J = 7.5 Hz, 2H), 1.66–1.56 (m, 2H), 1.40–1.19 (m, 31H), 0.87 (t, J = 6.8 Hz, 3H).
BCA-ODT: ^1^H NMR (400 MHz, Chloroform-d) δ6.86 (d, J = 2.2 Hz, 2H), 6.75 (d, J = 2.2 Hz, 2H), 2.68–2.63 (m, 4H), 1.70–1.64 (m, 4H), 1.57 (s, 6H), 1.51 (t, J = 7.5 Hz, 4H), 1.25 (m, 56H), 0.88 (t, J = 6.9 Hz, 6H).

From the ^13^C NMR spectrum of BCA-ODT (Figure 1E), a distinct signal at δ14.28 corresponded to the methyl carbon at the end of the long alkyl chain, serving as a characteristic signal for the adduct. Multiple peaks in the range from δ22.85 to δ42.16 were assigned to the methylene carbons of the alkyl chain derived from ODT as well as the saturated carbon atoms of the isopropyl group derived from the BPA backbone. In the downfield region, the signals between δ143.39 and δ115.53 were attributed to the aromatic carbon atoms of the benzene rings. Furthermore, the number of carbon resonances observed in the spectrum was consistent with the molecular symmetry, further confirming the successful synthesis of the target product.

The ^13^C NMR data are as follows: ^13^C NMR (101 MHz, Chloroform-d) δ143.39, 143.19, 142.14, 124.83, 118.41, 115.53, 42.16, 36.88, 34.21, 32.07, 30.91, 29.86, 29.86, 29.81, 29.75, 29.75, 29.68, 29.52, 29.52, 29.30, 29.23, 28.69, 28.53, 24.82, 22.85, 14.28.

Overall, the NMR results were consistent with the proposed molecular structure, indicating that the ODT was covalently coupled to the BQA through an aryl thioether bond to generate *S*-catecholyl functionalities. This result clearly and unambiguously demonstrated that oxidized BPA could be restored upon thiol addition.

**Figure 2:**
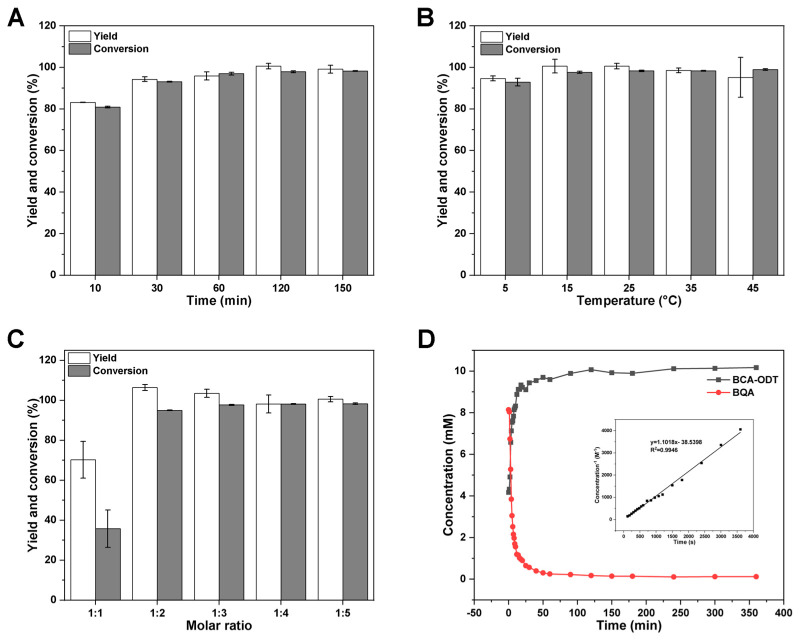
Synthesis of BCA-ODT. (**A**) Effects of reaction time on the yield and conversion of the product. (**B**) Effects of reaction temperature on the yield and conversion of the product. (**C**) Effects of the molar ratio of reactants on the yield and conversion of the product. (**D**) Time course. The inset presents the fitted second-order kinetic plot of the reaction.

Quantitative determination of BCA-ODT was performed using the standard curve at an absorbance of 291 nm (Figure S3B). Single-factor conditions were optimized to improve the yield of BCA-ODT (Figure 2A–C). A yield of 108.12% was obtained under the optimized conditions with a reaction time of 120 min, temperature of 25 °C, and molar ratio of BQA to ODT of 1:2. The UV-based yield may be slightly overestimated because minor byproducts formed during subsequent oxidation and side reactions can also contribute to the absorbance at 290 nm. A light yellow powder was obtained. A molar ratio of 1:2 gave the highest yield, which matched the reaction stoichiometry of thiol-catechol adduct formation, given that one BQA molecule possesses two o-quinone moieties. Consequently, catechol groups that participate in metal complexation and are susceptible to oxidation into o-quinone could be preserved in the form of thiol-catechol adducts while maintaining intact catechol functionality, thereby contributing to durable adhesion.

A kinetic study was conducted by monitoring the absorbance at 380 nm (Figure S5). The absorbance showed a remarkably rapid decay (Figure 2D). Half of the BQA was consumed within 2 min and disappeared completely within up to 2 h. Similar phenomena have been widely observed [15]. The reaction between thiol and o-quinone is reported to be one of the most favorable reactions [24]. The apparent reaction rate constant was approximately 1.11 M^−1^ s^−1^. Such effective coupling was possibly owing to the strong nucleophilicity of the thiol group in ODT [8]. Therefore, the strategy allowed for efficient preparation of biomimetic lubricants under mild conditions.

### 3.3. Adhesion Performance of Thiol-Functionalized Lubricant

**Figure 3:**
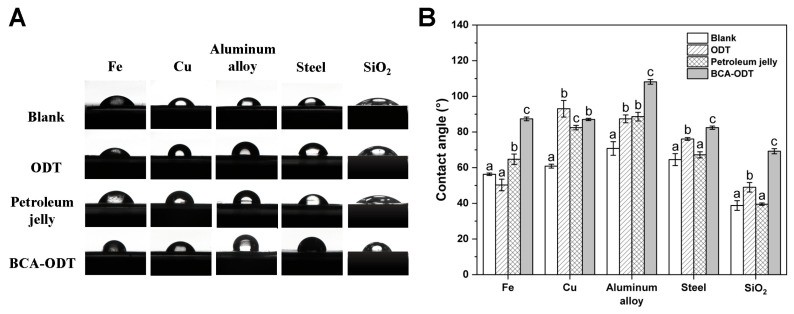
Adhesion performance of different lubricants on representative solid surfaces. (**A**) Representative contact angle images of water droplets on solid surfaces after treatment with different lubricants. Blank, ethanol. (**B**) Quantitative contact angle results corresponding to the images shown in panel A. Different lowercase letters indicate statistically significant differences among treatments for the same substrate based on ANOVA.

The formation of the lubricating film was further investigated by measuring the water contact angles of substrate sheets treated with different lubricants. As shown in Figure 3, both the metal sheets and glass sheets soaked in the BCA-ODT solution exhibited the largest contact angles among all tested metals. These results suggested that BCA-ODT significantly enhanced the surface hydrophobicity of the metal and glass substrates. In contrast, substrate sheets immersed in ODT solution or petroleum jelly, used as control samples, only showed a slight increase in contact angle compared with the blank sample. This observation revealed that ODT and petroleum jelly exhibited weak adhesion to the metal and glass surfaces, despite their inherent hydrophobicity. It should be noted that the relatively large contact angle observed for the copper sheet immersed in ODT solution may be attributed to the strong interaction between the thiol group of ODT and copper ions, which promotes adsorption on the copper surface. Catechol groups are known to form strong coordination interactions with metal and glass surfaces, providing significantly stronger adsorption than physical adsorption alone [7]. Therefore, BCA-ODT can firmly adhere to the metal and glass surface through its catechol moieties to form a stable adsorption film. Meanwhile, the outward-oriented long alkyl chains impart pronounced hydrophobicity, facilitating the formation of a lubricating layer that reduces friction and improves lubrication performance [25]. In addition, the presence of sulfur atoms may further enhance adsorption on metal surfaces, contributing to the stability of the lubricating film [21]. Since SiO_2_, the main component of glass, is also the dominant constituent of most common rocks, BCA-ODT can adhere not only to the metallic surface of drilling tools but also to the rock surfaces of wellbore walls to enhance the absorption strength of the lubricating film.

### 3.4. Metal Ion Interactions Ability of Thiol-Functionalized Lubricant

The adsorption capability of the synthesized product is primarily attributed to its catechol moieties, which can participate in multiple covalent and non-covalent interactions, thereby significantly enhancing adhesion at various interfaces. To elucidate the dominant interaction mechanism of the product and evaluate its potential applications, its coordination behavior with metal ions was investigated [26].

**Figure 4:**
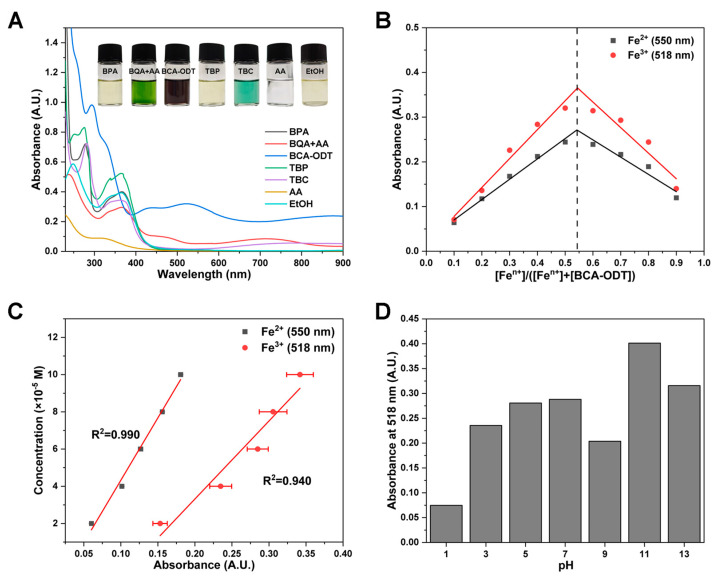
Coordination behavior of BCA-ODT toward iron ions. (**A**) Photographs and UV-Vis absorption spectra after reaction with Fe^3+^. BQA + AA, BQA reduced by ascorbic acid; TBP, tert-butylphenol; TBC, tert-butylcatechol; AA, ascorbic acid; EtOH, ethanol. (**B**) Job’s plots for the BCA-ODT complexes with Fe^2+^ and Fe^3+^, recorded at 550 and 518 nm, respectively. (**C**) Calculation of the conditional stability constants (K_cond_) for the BCA-ODT-Fe^2+^ and BCA-ODT-Fe^3+^ complexes. (**D**) Influence of pH on Fe^3+^ binding by BCA-ODT, as reflected by the absorbance at 518 nm.

Upon addition of Fe^3+^ ions, the color of the BCA-ODT solution changed from colorless to a deep purplish-brown solution, whereas the BPA solution remained unchanged (Figure S6). When BCA was obtained by reducing BQA with an excess of ascorbic acid, the addition of Fe^3+^ ions also produced a typical greenish coloration. Similar phenomena were observed for tert-butylphenol (TBP) and tert-butylcatechol (TBC), which served as model molecules for monophenol and diphenol structures, respectively. These results indicated that only compounds containing catechol moieties exhibited visible color changes upon coordination with Fe^3+^ ions, whereas compounds possessing monophenol moieties showed no such response. As shown in the UV-Vis spectra (Figure 4A), a new absorption band appeared at 550 nm after the interaction between BCA-ODT and Fe^3+^ ions [27]. Coordination between the central metal ion and the ligand expands the electron cloud distribution, leading to a bathochromic shift of the characteristic absorption band of the complex. The characteristic absorption confirmed the coordination ability of BCA-ODT toward metal ions arising from the catechol moiety. Fe^3+^ ions can form mono-, bis-, and tris-catecholate complexes depending on the pH and the ligand-to-metal ratio. These complexes typically exhibit characteristic ligand-to-metal charge transfer absorption bands, giving rise to distinctive colors in the visible region. It is generally believed that mono-catecholate complexes are green, while bis-catecholate complexes are purple. Such color differences implied that BCA-ODT exhibited a bis-coordination environment with Fe^3+^ ions [28].

Job’s plots are widely employed to investigate complex stoichiometry and coordination behavior [29]. BCA-ODT exhibited an apparent 1:1 complexation stoichiometry with both Fe^3+^ and Fe^2+^. Given that the BCA-ODT molecule contains two catechol functional groups, a single metal ion could simultaneously coordinate with two catechol moieties, resulting in an intrinsic 1:2 (metal ion: catechol) binding mode to form bis-coordination. This conclusion was consistent with the color reaction results of BCA-ODT and Fe^3+^ (Figure 4B). Bis-coordination shows a higher affinity and longer lifetime than mono-coordination [30,31]. Considering the structure and steric hindrance of BCA-ODT, it is unlikely that the two catechol groups from a single BCA-ODT molecule simultaneously coordinate with one iron ion. Instead, it is reasonable to hypothesize that one iron ion separately coordinates with one catechol group from each of two different BCA-ODT molecules, thereby forming a metal-catechol cross-linking structure. Such a putative multidentate coordination network is conducive to enhancing the cohesion strength of the lubricant molecules, and improving the adsorption strength and stability of the interfacial film. By contrast, conventional lubricants mainly depend on weak single-point adsorption, which was easily disrupted. These findings suggested that the enhanced metal ion coordination interaction might be one of the key reasons for the superior lubricating and anti-wear properties of the product in water-based drilling fluids. Utilization of BPA to generate biscatechol is therefore crucial for the formation of a plausible cross-linking coordination network. The coordination with Fe^3+^ was barely affected within the pH range from 3 to 11, demonstrating that the product possesses a wide pH tolerance for potential applications (Figure 4D and Figure S7).

The modified Benesi–Hildebrand (B-H) method was employed to determine the conditional stability constants (K_cond_) of the complexes [23]. The log (K_cond)_ values for BCA-ODT were 4.09 and 5.02 M^−1^ for Fe^3+^ and Fe^2+^, respectively (Figure 4C and Figure S8). BCA-ODT exhibited higher complexation stability toward Fe^2+^ than toward Fe^3+^, which may be attributed to differences in the electronic configurations of the two iron oxidation states and their coordination modes with the catechol groups.

These results indicated that BCA-ODT possesses strong coordination capability and favorable affinity toward both Fe^3+^ and Fe^2+^ ions, confirming that the introduction of catechol moieties into the molecular structure effectively enhanced interactions with iron ions [32]. This coordination mechanism provides a theoretical basis for its application as a drilling fluid lubricant and highlights its potential advantages in drilling operations.

### 3.5. Autoxidation Resistance and Thermal Stability of Thiol-Functionalized Lubricant

**Figure 5:**
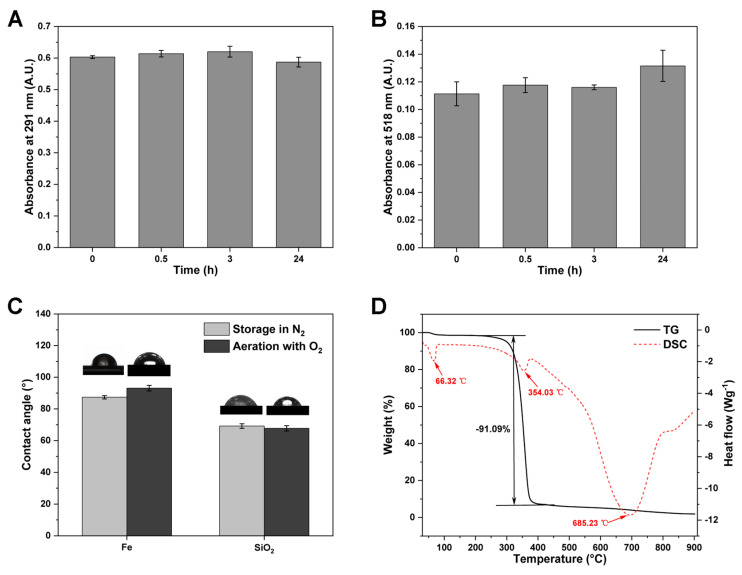
Stability of BCA-ODT. (**A**) Variation in the characteristic UV-Vis absorbance of BCA-ODT at 291 nm after O_2_ aeration for different times. (**B**) Variation in the characteristic absorbance at 518 nm of the BCA-ODT-Fe^3+^ complex formed by samples aerated with O_2_ for different times. (**C**) Comparison of the contact angles of BCA-ODT stored under N_2_ and after O_2_ aeration for 24 h on iron and SiO_2_ surfaces. The photos of contact angles are positioned above the column chart. (**D**) TG and DSC curves of BCA-ODT.

Upon aeration with oxygen for 24 h, the absorbance of the characteristic peak at 291 of BCA-ODT remained nearly unchanged, indicating that the catechol structure in BCA-ODT exhibited good resistance toward autoxidation (Figure 5A). Furthermore, the coordination ability to Fe^3+^ showed no significant decrease after exposure to oxygen for 24 h (Figure 5B). Consistently, the change in the water contact angle on metal and glass surfaces treated with BCA-ODT after 24 h of air oxidation was negligible, indicating that the adhesive strength and lubrication performance of the lubricating film were well preserved, which further demonstrated the excellent oxidative stability of BCA-ODT (Figure 5C).

The improved oxidative stability can be attributed to the thiol functionalization introduced into the molecular structure, which protects the reduced form of catechol from autoxidation. The long alkyl chain linked on ortho-position through the thioether bond introduces steric hindrance, forming a physical barrier that limits the approach of oxygen molecules and reactive radicals to the catechol hydroxyl groups. Furthermore, the sulfur atom may act as a radical scavenger, suppressing the propagation of oxidation reactions. The presence of sulfur can also exert an electron-withdrawing inductive effect, slightly decreasing the electron density of the phenolic hydroxyl groups and thereby reducing the tendency for dehydrogenation and free radical formation. These combined effects contribute to the enhanced oxidative stability of BCA-ODT, enabling it to maintain stable adhesion and lubrication performance even after prolonged oxidation.

As shown in Figure 5D, thermogravimetric analysis (TG) and differential scanning calorimetry (DSC) were employed to evaluate the thermal stability of the product. The thermal degradation of BCA-ODT exhibited three distinct stages. The first stage occurred between 47 and 67 °C with a mass loss of 1.76%, accompanied by a small endothermic peak, which was attributed to the melting of the product and the removal of residual moisture. The second stage occurred between 250 and 400 °C with a mass loss of 91.09%, and the maximum degradation peak was observed at 359.37 °C, corresponding to the thermal decomposition and chain scission of the product. The third stage occurred above 400 °C, where the product gradually lost mass (5.27%) and exhibited a broad endothermic peak, indicating further carbonization and decomposition of the residual structure. In contrast, the thermal decomposition of ODT occurred near 265.16 °C (Figure S9). These results suggested that the rigid aromatic ring structures significantly enhanced the thermal stability of the product. Below 250 °C, the product exhibited excellent thermal stability, indicating its potential for high-temperature applications.

### 3.6. Lubrication Performance of Thiol-Functionalized Lubricant

**Figure 6:**
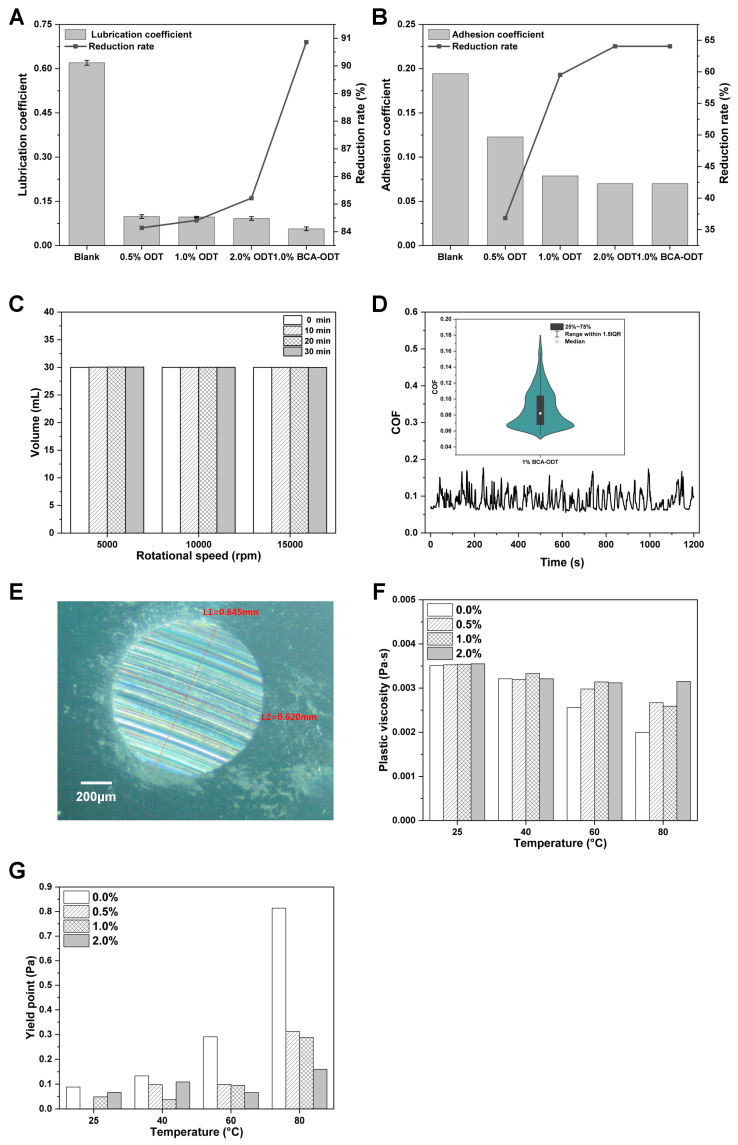
Evaluation of the lubrication-related properties of BCA-ODT. (**A**) Lubrication coefficient of drilling fluids containing different additives. (**B**) Adhesion coefficient for filter-cake adhesion. (**C**) Foaming behavior of the drilling fluids under different stirring speeds and times. (**D**) Time-dependent COF curve of BCA-ODT in the friction test, with the corresponding COF distribution shown in the insets. (**E**) Corresponding wear scar image. (**F**) Plastic viscosity derived from Bingham plastic model fitting of the rheological curves. (**G**) Yield point derived from Bingham plastic model fitting of the rheological curves.

The extreme pressure lubrication performance of BCA-ODT at a 1% dosage in sodium bentonite fluid was evaluated. The lubrication coefficient was measured to be 0.06, showing a 90.86% reduction compared to the blank base slurry. Furthermore, the mud cake adhesion coefficient was measured to be 0.07 (Figure 6A,B). BCA-ODT significantly lowered the mud cake adhesion coefficient by 64.04% when compared to the base fluid, indicating its effective role in improving lubrication. The enhanced lubrication performance can be attributed to the catechol moiety, which forms strong coordination bonds with metal surfaces, thereby strengthening the lubricating film and effectively resisting shear forces from clay particles. Additionally, sulfur elements, acting as extreme-pressure lubrication groups, are likely responsible for reducing friction and wear under high loads by forming a protective sulfide layer on the metal surfaces. This layer reduces direct metal-to-metal contact, minimizing wear under extreme pressure conditions [21].

The volume of sodium bentonite fluid appeared to remain unchanged under high stirring at 15,000 rpm for 30 min in the presence of BCA-ODT at a 1% dosage (Figure 6C). BCA-ODT did not generate foam, which can be attributed to its low hydrophilic-lipophilic balance (HLB) value of 1.30 [3]. The low HLB indicated a greater affinity for oil phases, thus reducing the tendency to form stable foams. Substances with an HLB value in the range of 1–3 are generally used as defoaming agents, because they tend to exist as tiny oil droplets in aqueous systems to destroy foam by reducing the surface viscosity and elasticity of the foam liquid film. Therefore, BCA-ODT is able to help maintain the constant density of the drilling fluid and improve the quality of the mud cake by enhancing the fluid’s stability and consistency.

The tribological performance of the drilling fluid was evaluated by the four-ball friction test. The coefficient of friction (COF) was recorded in real time. With the addition of 1% BCA-ODT, the COF remained stable at approximately 0.09 throughout the test period, exhibiting slight fluctuations. The low COF demonstrated the effective friction-reducing capability of BCA-ODT. No distinct initial running-in stage was observed, which could be attributed to rapid formation of lubricating film due to the quick kinetics of the complexation between the catechol moiety and iron ions. The minor fluctuations in COF were ascribed to the dynamic equilibrium between film wear and in situ reassembly. Therefore, BCA-ODT enables an immediate lubrication state for effective protection within an extremely short time. The wear morphology of the steel ball surfaces was characterized after the friction test. As shown in Figure 6D,E, the wear scars formed in the presence of BCA-ODT exhibited relatively shallow ploughing furrows with uniform and well-defined scratch patterns, suggesting a mild abrasive wear mechanism. The BCA-ODT system has a relatively small wear scar diameter (WSD) of 0.63 mm. Although the WSD value of BCA-ODT was larger than that of the synthetic ester-type catechol-based lubricant, their COF values were relatively close [3]. BCA-ODT formed lubricating films on the steel ball friction surfaces with good wear resistance for better protection.

The rheological characteristics of drilling fluids, which determine their capacity to transport and suspend drill cuttings, are critical parameters for the design and evaluation of drilling fluid systems [33]. Rheological measurements were conducted at representative temperatures of 25, 40, 60, and 80 °C to investigate the temperature-dependent behavior of the lubricants. As shown in Figure S10, the addition of BCA-ODT at different dosages did not significantly alter the pseudoplastic characteristics of the drilling fluid, which exhibited typical shear-thinning behavior. At 80 °C, both the shear stress and apparent viscosity of the drilling fluid decreased after the addition of BCA-ODT at a given shear rate, suggesting that the lubricant reduced the internal friction within the fluid system. This phenomenon may be attributed to the enhanced molecular mobility and improved dispersion of BCA-ODT at elevated temperatures, which could facilitate the lubrication effect and weaken the interactions between solid particles in the drilling fluid.

The rheological parameters of the drilling fluids were further evaluated using the Bingham plastic model (Figure 6F,G). The calculated plastic viscosity (PV) of the system remained relatively low, approximately 0.002–0.004 Pa·s, over the investigated temperature range, indicating reduced internal friction within the fluid system [34]. Meanwhile, the yield point (YP) was maintained at approximately 0–1 Pa, suggesting that the incorporation of BCA-ODT did not significantly alter the overall structural framework formed by bentonite particles in the base fluid. The relatively high YP/PV ratio implied that the drilling fluid retained a structure-sensitive rheological response, which was beneficial for maintaining suspension capacity under low-shear conditions. At the same time, the low PV contributes to reduced flow resistance at elevated shear rates, thereby facilitating efficient cuttings transport during circulation. These results demonstrated that the addition of BCA-ODT provides lubrication functionality while preserving the essential rheological characteristics required for drilling fluid performance [35].

BCA-ODT exhibited strong adhesion to metal surfaces due to the presence of catechol functional groups, which are known to form robust coordination interactions with metal substrates. As a result, BCA-ODT can form a compact and stable lubricating film on the contact surfaces. This protective layer effectively separates the drill string from the wellbore wall, thereby preventing direct solid–solid contact and reducing friction. Consequently, the overall lubrication performance of the drilling fluid is significantly improved.

## 4. Conclusions

In this study, inspired by mussel adhesion and melanogenesis, an innovative synthetic strategy for a drilling fluid lubricant was developed to overcome the instability of catechol. Taking advantage of the high reactivity of thiol-quinone Michael addition, a catechol-based biomimetic lubricant (BCA-ODT) was successfully synthesized from BPA-derived quinone and ODT. The molecular structure contains both catechol moieties and long alkyl chains, where the former provides strong interfacial adhesion while the latter imparts excellent hydrophobicity.

Compared with the existing catechol-based lubricants, this strategy exhibits four distinct advantages. (i) Autoxidation resistance via thiol functionalization: The formation of an *S*-catecholyl linkage endowed the molecule with enhanced resistance to catechol autoxidation and helped preserve the active catechol functionality. (ii) Cross-linking coordination network via bis-coordination: The biscatechol groups can form a coordination network with metal ions, promoting the formation of a dense and stable lubricating film on metal surfaces to enhance adhesion strength. (iii) Introduction of sulfur as an extreme pressure element: Sulfur atoms further improved the extreme-pressure lubrication performance. BCA-ODT exhibited good thermal stability up to 350 °C, indicating its suitability for high-temperature drilling environments. (iv) Thiol-quinone coupling reaction: The coupling reaction proceeds rapidly under mild conditions, enabling a facile and efficient synthesis.

The thiol-functionalized biomimetic lubricant integrates bioinspiration from both melanin biosynthesis and mussel adhesion. It features novel structure analogous to melanin with *S*-catecholyl linkage. Notably, the thiol-functionalized lubricant enables a more accurate mimicry of mussel adhesion. It not only emulates the adhesion mechanism of Mfp-3 and Mfp-5, but also, for the first time, further imitates the protective mechanism of Mfp-6, which shields catechol groups from oxidative degradation and preserves their adhesive capability [8]. ODT serves as the critical material in this study for realizing the proposed innovative biomimetic concepts.

Overall, the developed biomimetic lubricant demonstrates strong resistance to autoxidation, excellent thermal stability, high adhesion capability, and effective lubrication performance, showing considerable potential for application in drilling fluid lubrication. The thiol-quinone coupling strategy provides a versatile and mild approach for the design of catechol-based biomimetic lubricants.

## Data Availability

The raw data supporting the conclusions of this article will be made available by the authors on request.

## Supplementary Materials

The following supporting information can be downloaded at https://www.mdpi.com/article/10.3390/biomimetics11040269/s1: Figure S1: Appearance and morphology of BQA and BCA-ODT; Figure S2: ^1^H NMR spectra of the starting materials and intermediate; Figure S3: Standard calibration curves used for the quantitative determination of BQA and BCA-ODT; Figure S4: Single-factor optimization of the reaction conditions for BQA synthesis; Figure S5: Kinetic fitting of the reaction process using different reaction-order models; Figure S6: Representative photographs and UV-Vis absorption spectra; Figure S7: Effect of pH on the interaction between BCA-ODT and Fe^3+^; Figure S8: UV-Vis absorption spectra used for the determination of the conditional stability constants (*K*_cond_) of the BCA-ODT complexes with iron ions; Figure S9: Thermal analysis of ODT and BCA-ODT; Figure S10: Rheological behavior of drilling fluids containing different dosages of BCA-ODT at different temperatures.
