# Supplementary material for: Melanin-Inspired Biomimetic Strategy for Preserving Adhesion of Lubricants via Thiol-Quinone Addition
Source: Biomimetics (Basel). 2026 Apr 14;11(4):269. doi: 10.3390/biomimetics11040269 (PMC13113061; doi:10.3390/biomimetics11040269)
Supplement: Supplementary file 1 [file biomimetics-11-00269-s001.zip › Supporting Information.pdf]

Supporting Information

Melanin-Inspired Biomimetic Strategy for Preserving Adhesion of Lubricants via Thiol-quinone Addition

Xiao Song[1], Chao Mei[2], Yinna Wu[1], Dan He[3], Junwei Zhu[1], Qi Chen[2], Jiaxin Guo[1], Zhengwei Zhao[2], Tonghui Xie[1], Wenbin Liu[1]*

[1] School of Chemical Engineering, Sichuan University, Chengdu 610065, China.

[2] Exploration Division, Southwest Oil & Gas Field Company, PetroChina, Chengdu 610041, China

[3] Oil & Gas Technology Research Institute, Southwest Oil & Gas Field Company, PetroChina, Chengdu 610017, China

* Corresponding author. Email address: wbliu@scu.edu.cn

ORCID ID: 0000-0002-3780-9394

Table of contents

Materials and Methods	S3
Figure S1. Appearance and morphology of BQA and BCA-ODT.	S8
Figure S2. ^{1}H NMR spectra of the starting materials and intermediate.	S9
Figure S3. Standard calibration curves used for the quantitative determination of BQA and BCA-ODT.	S10
Figure S4. Single-factor optimization of the reaction conditions for BQA synthesis.	S11
Figure S5. Kinetic fitting of the reaction process using different reaction-order models.	S12
Figure S6. Representative photographs and UV-Vis absorption spectra.	S13
Figure S7. Effect of pH on the interaction between BCA-ODT and Fe^{3+}.	S14
Figure S8. UV-Vis absorption spectra used for the determination of the conditional stability constants (K_{cond}) of the BCA-ODT complexes with iron ions.	S15
Figure S9. Thermal analysis of ODT and BCA-ODT.	S16
Figure S10. Rheological behavior of drilling fluids containing different dosages of BCA-ODT at different temperatures.	S17

MATERIALS AND METHODS

1. Establishment of the BQA Calibration Curve

To establish the calibration curve, a series of standard solutions of BQA with known concentrations of 10, 20, 30, 40 and 50 mg · L^{-1} were prepared using dichloromethane. The absorbance values were measured at 380 nm using a Mapada UV-3100PC spectrophotometer (Mapada Instruments, Shanghai, China) equipped with a quartz cuvette with a path length of 10 mm. Dichloromethane was used as the blank control.

The calibration curve was constructed by plotting absorbance against concentration, and linear regression analysis was performed to obtain the calibration equation. The resulting regression equation was $y = 0.0033x + 0.0034$, with a correlation coefficient (R^2) of 0.9994. Each measurement was performed in triplicate, and the average value was used for calibration.

The established calibration curve was subsequently used for the quantitative determination of BQA in the crude product.

2. Establishment of the BCA-ODT Calibration Curve

To establish the calibration curve, a series of standard solutions of BCA-ODT with known concentrations of 10, 20, 30, 40, 50, 75, 100, 150 and 200 mg·L^{-1} were prepared using dichloromethane. The absorbance values were measured at 291 nm using a Mapada UV-3100PC spectrophotometer (Mapada Instruments, Shanghai, China) equipped with a quartz cuvette with a path length of 10 mm. Dichloromethane was used as the blank control.

The calibration curve was constructed by plotting absorbance against concentration, and linear regression analysis was performed to obtain the calibration equation. The resulting regression equation was $y = 0.0062x - 0.0152$, with a correlation coefficient (R^2) of 0.9990. Each measurement was performed in triplicate, and the average value was used for calibration.

The established calibration curve was subsequently used for the quantitative determination of BCA-ODT in the crude product.

3. Single-factor optimization of the BQA reaction conditions

Bisphenol A (BPA, 228.29 mg, 1 mmol) was dissolved in methanol (10 mL), followed by the addition of 2-iodoxybenzoic acid (IBX, 280.02 mg, 1 mmol). The mixture was magnetically stirred in a water bath at 25 °C for 15 min. After completion of the reaction, the mixture was subjected to vacuum filtration, and the obtained solid was air-dried at room temperature. The crude product was dissolved in dichloromethane and centrifuged to remove insoluble impurities. The supernatant was concentrated under reduced pressure to afford the BQA solid.

Based on the above initial conditions, systematic optimization of key reaction parameters was carried out to improve the reaction efficiency, including the molar ratio of oxidant to substrate, the initial concentration of BPA, the reaction temperature, and time. After drying, the crude product was weighed to obtain m_{Total}. Subsequently, 5 mg of the crude product was dissolved in 2 mL of dichloromethane, centrifuged, and the supernatant was diluted 100-fold. The absorbance of the solution at 380 nm was measured using a Mapada UV-3100PC spectrophotometer (Mapada Instruments, Shanghai, China) equipped with a quartz cuvette with a path length of 10 mm. The same post-treatment procedure was applied after each reaction under different experimental conditions.

According to the calibration curve of BQA, the ratio m_{BQA}/m_{Total} was calculated using Equation (S1), and the yield of BQA was calculated using Equation (S2). By comparing the yield and the value of m_{BQA}/m_{Total} under different reaction conditions, the optimal reaction parameters were determined.

$$m_{BQA}/m_{Total} = \frac{A - 0.0034}{0.013 \times 25} \times 100\% \qquad (S1)$$

$$Y = \frac{(A - 0.0034) \times m_{Total}}{0.013 \times 25 \times m_{Theoretical}} \times 100\% \qquad (S2)$$

where A represents the measured absorbance at 380 nm; m_{BQA}/m_{Total} represents the mass fraction of BQA in the crude product; Y represents the yield of BQA; m_{Total} is the mass

of the dried crude product (mg); and $m_{Theoretical}$ represents the theoretical yield of BQA under the corresponding reaction conditions (mg).

4. Single-factor optimization of the BCA-ODT reaction conditions

BQA (51.25 mg, 0.2 mmol) was dissolved in dichloromethane (15 mL), while 1-octadecanethiol (ODT, 286.56 mg, 1 mmol) was dissolved in dichloromethane (5 mL). The two solutions were mixed and stirred in a water bath at 25 °C for 2.5 h. The reaction was conducted under a nitrogen atmosphere to prevent the formation of disulfide bonds between thiol groups. After completion, the reaction mixture was concentrated under reduced pressure to afford a crude product, which was purified by column chromatography.

Based on the above initial conditions, systematic optimization of the key reaction parameters was conducted to improve the reaction efficiency, including the reaction time, reaction temperature, and the molar ratio of BQA to ODT. After completion of the reaction, the reaction mixture was diluted 50-fold, and the absorbance values at 291 nm and 380 nm were measured using a Mapada UV-3100PC spectrophotometer (Mapada Instruments, Shanghai, China) equipped with a quartz cuvette with a path length of 10 mm. The same post-treatment procedure was applied to all reactions conducted under different conditions.

According to the calibration curve of the product, the yield of the lubricant product was calculated using Equation (S3), and the conversion of BQA was determined using Equation (S4). By comparing the yield and conversion values under different reaction conditions, the optimal reaction parameters were determined.

$$Y = \frac{A_{291} + 0.01516}{0.00743 \times m_{Theoretical}} \times 100\% \qquad (S3)$$

$$C = 1 - \frac{A_{380} - 0.0034}{0.013 \times m_{BQA\ initial}} \times 100\% \qquad (S4)$$

where Y represents the yield of the lubricant product; A_{291} and A_{380} represent the absorbance values at 291 nm and 380 nm, respectively; $m_{Theoretical}$ represents the

theoretical mass of the product under the given reaction conditions (mg); *C* denotes the conversion of BQA; and $m_{BQA\ initial}$ represents the initial mass of BQA before the reaction (mg).

5. Interaction with metal ions

Equal amounts of PTBP, TBC, BPA, BQA, and BCA-ODT were separately dissolved in anhydrous ethanol to prepare solutions with the same mass concentration. A 100 μM ascorbic acid aqueous solution and a 10 mM ferric chloride ethanol solution were also prepared. The solutions were transferred into sample vials and photographed to record any color changes. The solutions were then appropriately diluted, and the UV-Vis absorption spectra were measured using a Mapada UV-3100PC spectrophotometer (Mapada Instruments, Shanghai, China) with a quartz cuvette having a 10 mm path length.

Additionally, a BQA and ascorbic acid mixture was pre-prepared. Then, equal volumes of ferric chloride solution were added to the PTBP, TBC, BPA, BCA-ODT, ascorbic acid, and BQA/ascorbic acid mixture systems. The systems were allowed to mix thoroughly, and the resulting solutions were again transferred to sample vials for photographing. After appropriate dilution, the UV-Vis spectra were recorded. By comparing the spectral changes before and after the addition of iron ions, the interaction characteristics of the different compounds and their complexes with iron ions were systematically analyzed.

6. Effect of pH on the coordination behavior between BCA-ODT and iron ions

BCA-ODT solutions (0.1 mM) were prepared in water/ethanol (1:4, v/v), and the pH values were adjusted to 1, 3, 5, 7, 9, 11, and 13 using hydrochloric acid and sodium hydroxide solutions. Each BCA-ODT solution was then mixed with an equal volume of $FeCl_3$ solution at a 1:1 molar ratio. After incubation at room temperature for 1 h to allow the coordination reaction to reach equilibrium, the full UV-vis spectra of the resulting mixtures were recorded using a Mapada UV-3100PC spectrophotometer (Mapada Instruments, Shanghai, China) with a quartz cuvette having a 10 mm path length.

7. Foaming performance test

Sodium bentonite fluid (Na-BT) with 1% BCA-ODT is prepared. The aged volume is first measured using a graduated cylinder, followed by stirring at 5000, 10000, and 15000 rpm for 10, 20, and 30 minutes. After stirring, the volume is measured. Measurements were repeated three times.

8. Calculation of HLB

The hydrophile-lipophile balance(HLB) value was given by Davies' method, equation as follows:

$$HLB = 7 + \sum(hydrophlilc\ group\ numbers) + \sum(lipophilic\ group\ numbers)\ (S5)$$

FIGURES

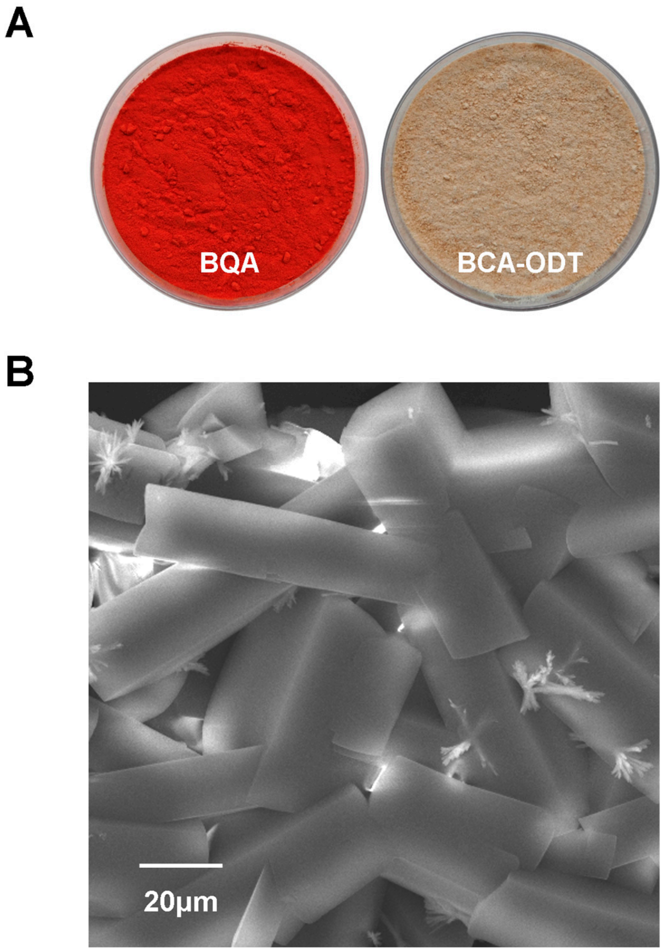

Figure S1. Appearance and morphology of BQA and BCA-ODT. (A) Photographs showing the macroscopic appearance of BQA and BCA-ODT. (B) SEM image of BQA, showing its surface morphology. Scale bar: 20 μm.

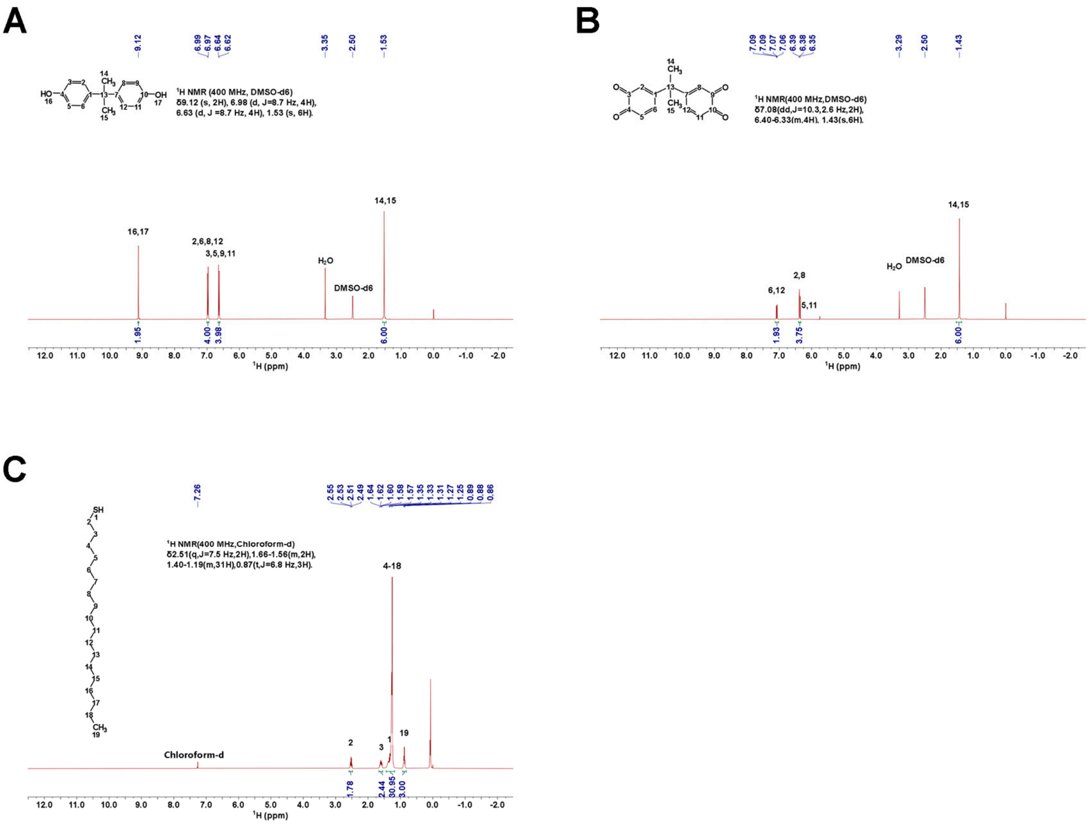

Figure S2. ${}^{1}H$ NMR spectra of the starting materials and intermediate. (A) ${}^{1}H$ NMR spectrum of BPA. (B) ${}^{1}H$ NMR spectrum of BQA. (C) ${}^{1}H$ NMR spectrum of ODT.

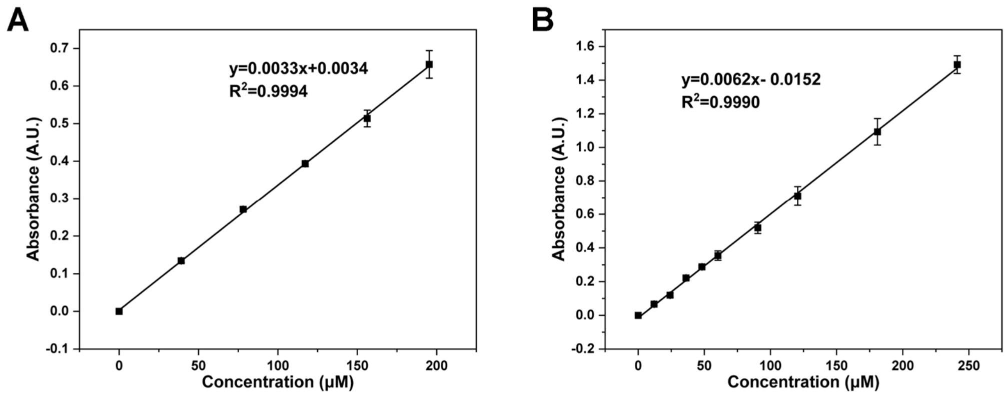

Figure S3. Standard calibration curves used for the quantitative determination of BQA and BCA-ODT. (A) Calibration curve of BQA. (B) Calibration curve of BCA-ODT. The fitted equations and corresponding correlation coefficients are shown in each panel.

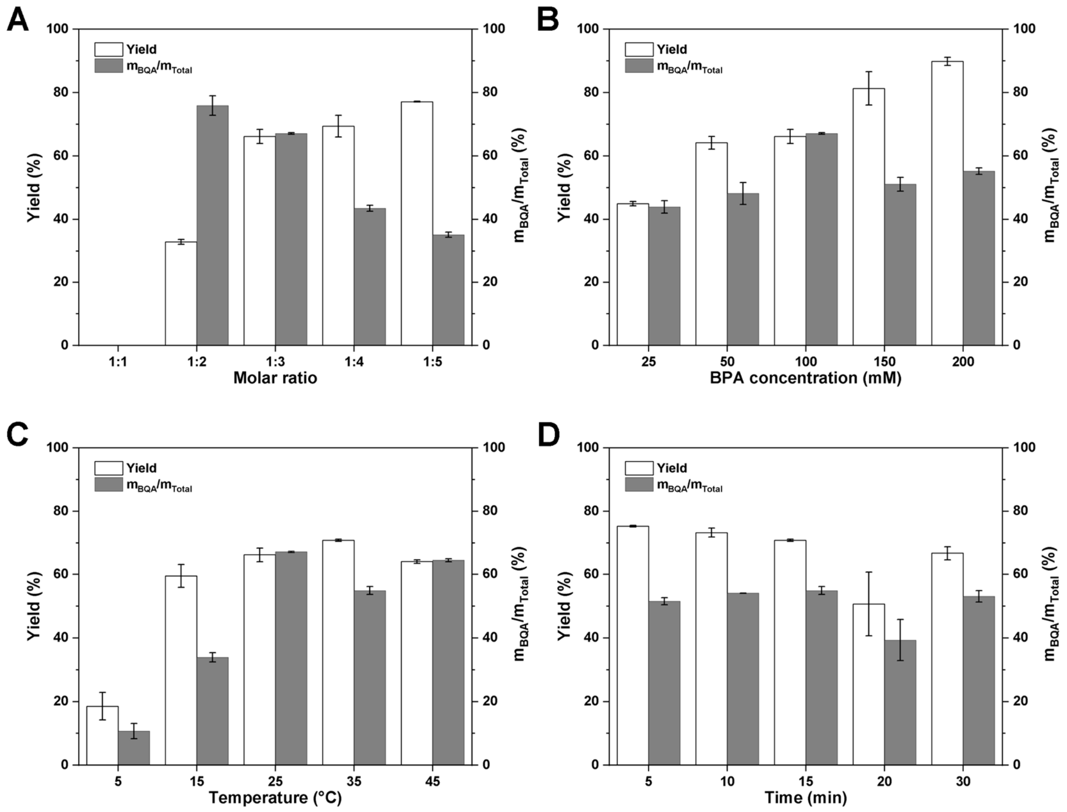

Figure S4. Single-factor optimization of the reaction conditions for BQA synthesis. (A) Effect of the molar ratio of reactants on the yield of BQA and the mass fraction of BQA in the total products (m_{BQA}/m_{Total}). (B) Effect of BPA concentration on the yield of BQA and m_{BQA}/m_{Total}. (C) Effect of reaction temperature on the yield of BQA and m_{BQA}/m_{Total}. (D) Effect of reaction time on the yield of BQA and m_{BQA}/m_{Total}.

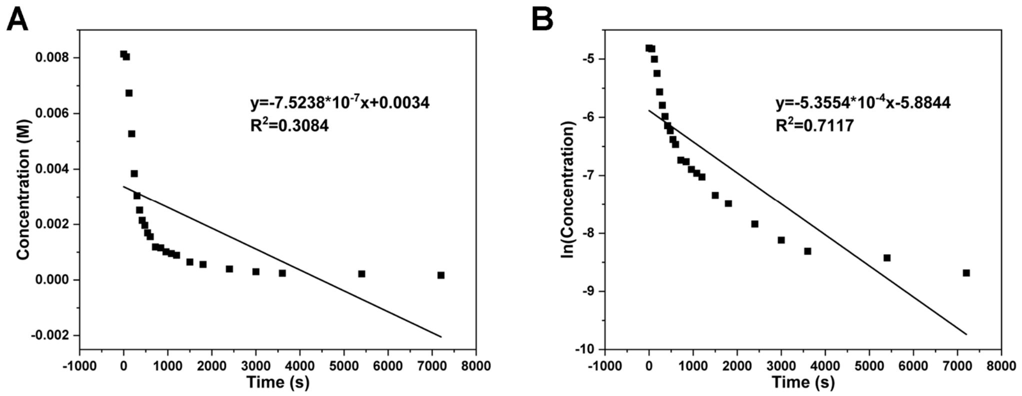

Figure S5. Kinetic fitting of the reaction process using different reaction-order models. (A) Zero-order kinetic fitting plot of concentration versus time. (B) First-order kinetic fitting plot of ln(concentration) versus time. The corresponding fitted equations and correlation coefficients (R^2) are shown in each panel.

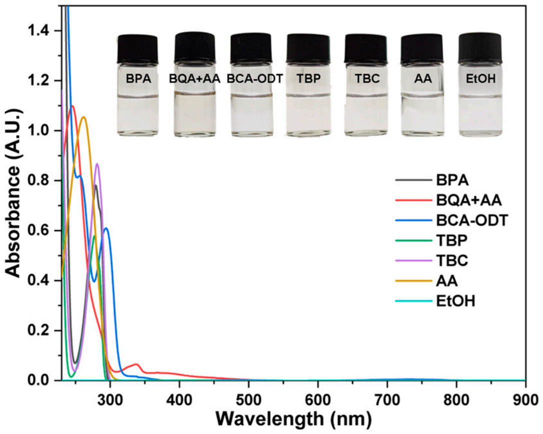

Figure S6. Representative photographs and UV-Vis absorption spectra. The inset shows the corresponding photographs of the samples.

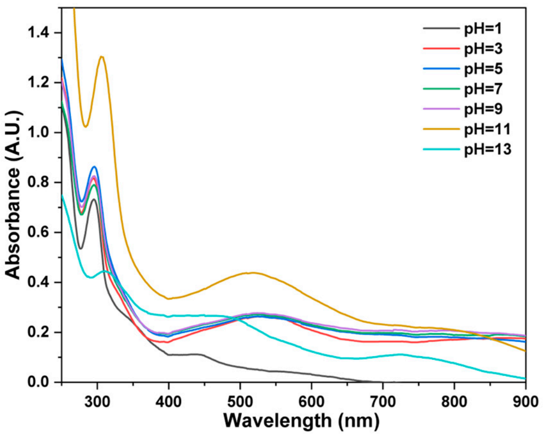

Figure S7. Effect of pH on the interaction between BCA-ODT and Fe^{3+}.

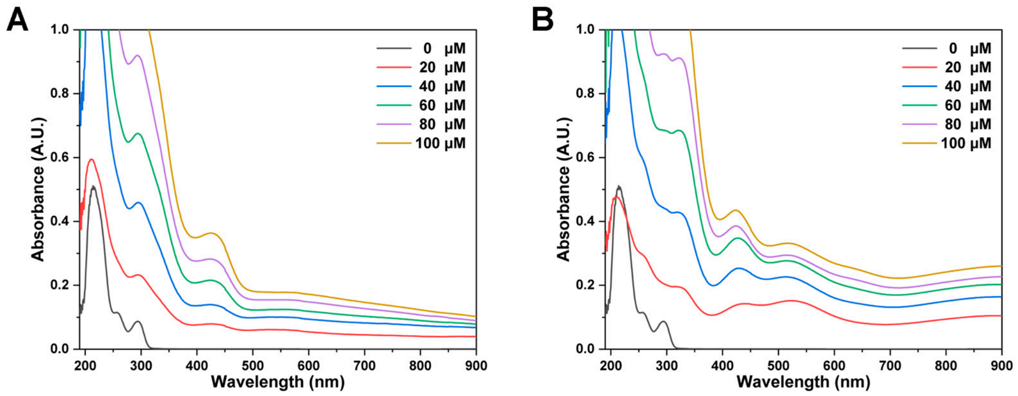

Figure S8. UV-Vis absorption spectra used for the determination of the conditional stability constants (K_{cond}) of the BCA-ODT complexes with iron ions. (A) UV-Vis spectra of the BCA-ODT-Fe^{2+} system recorded at different Fe^{2+} concentrations (0-100 μM). (B) UV-Vis spectra of the BCA-ODT-Fe^{3+} system recorded at different Fe^{3+} concentrations (0-100 μM).

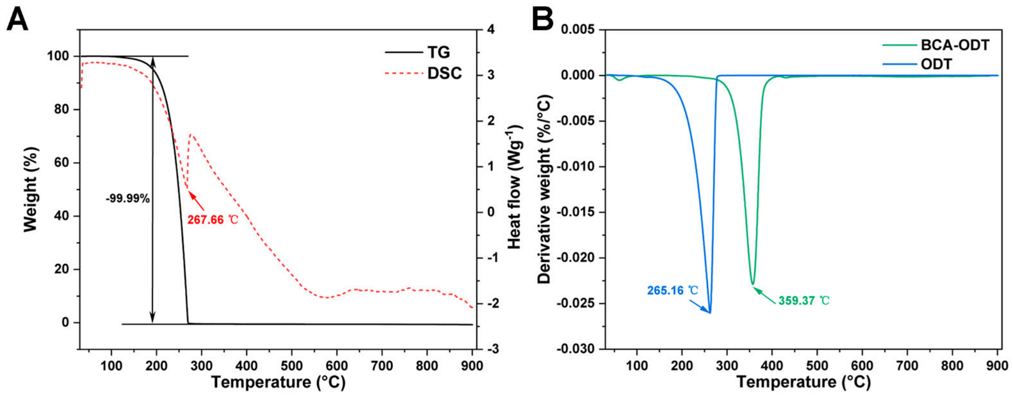

Figure S9. Thermal analysis of ODT and BCA-ODT. (A) TG and DSC curves of ODT. (B) DTG curves of BCA-ODT and ODT.

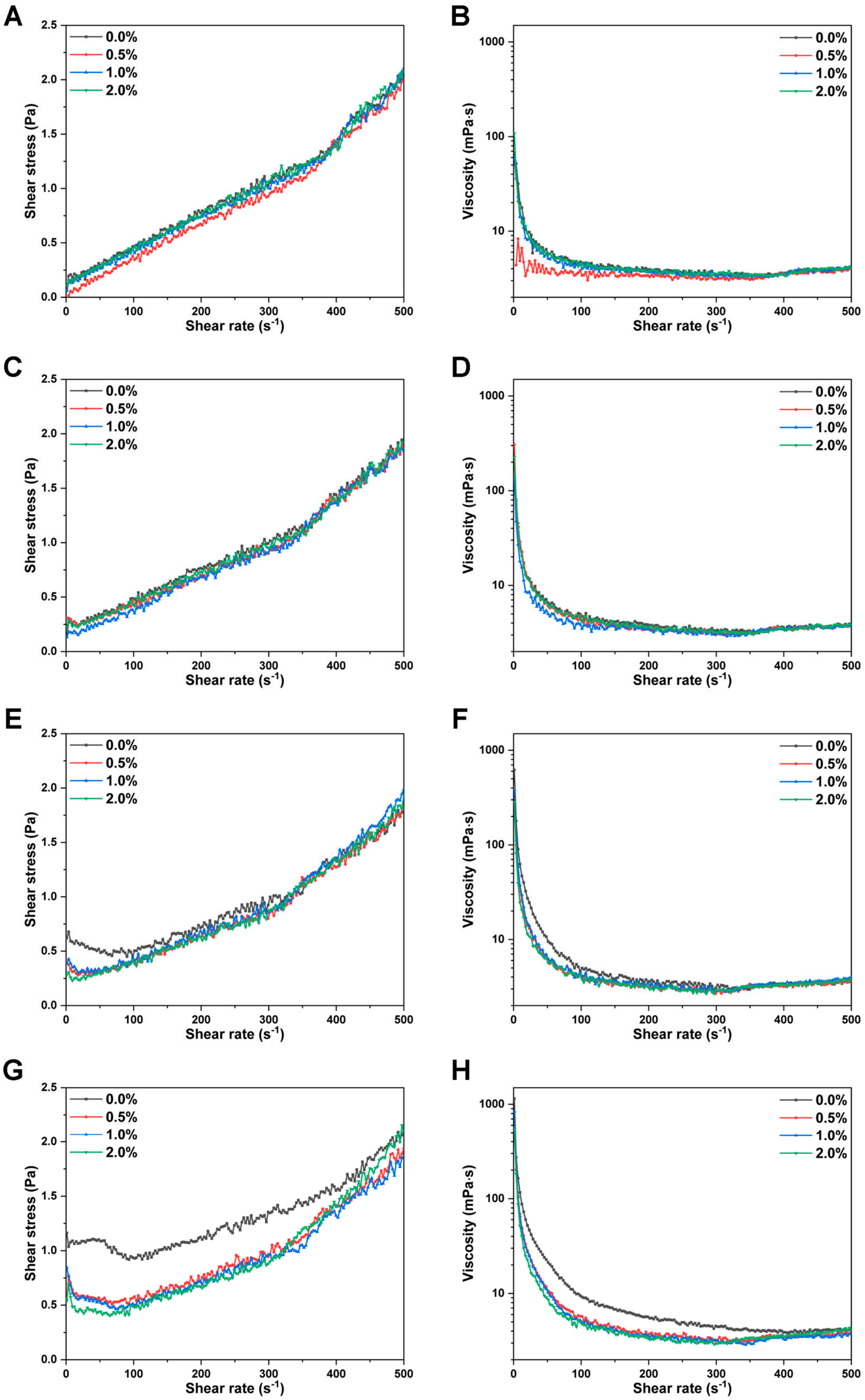

Figure S10. Rheological behavior of drilling fluids containing different dosages of BCA-ODT at different temperatures. (A,C,E,G) Shear stress–shear rate curves of the

drilling fluids containing 0, 0.5%, 1.0%, and 2.0% BCA-ODT at 25, 40, 60, and 80 °C, respectively. (B,D,F,H) Corresponding apparent viscosity–shear rate curves at 25, 40, 60, and 80 °C, respectively. The rheological profiles were used to evaluate the effects of BCA-ODT dosage and temperature on the flow behavior of the drilling fluids.
